# Masamune: a tool for automatic dynamic PET data processing, image reconstruction and integrated PET/MRI data analysis

**DOI:** 10.1186/2197-7364-1-S1-A57

**Published:** 2014-07-29

**Authors:** Daniel B Chonde, David Izquierdo-Garcia, Kevin Chen, Spencer L Bowen, Ciprian Catana

**Affiliations:** Athinoula A. Martinos Center for Biomedical Imaging, Department of Radiology, Massachusetts General Hospital and Harvard Medical School, Charlestown, MA USA; Department of Health Sciences and Technology, Massachusetts Institute of Technology, Cambridge, MA USA; Program in Biophysics, Harvard University, Cambridge, MA USA

We describe a novel semi-automated pipeline which integrates advanced data analysis tools for MR and PET with advanced PET reconstruction correction methods (partial volume effect correction [PVC], motion correction [MC], attenuation correction [AC]) in a user-friendly Matlab graphical user interface (GUI).

The reconstruction and analysis GUI is written in Matlab. Computationally intensive tasks in the pipeline are automatically transferred to a high-performance computing cluster and retrieved.

Descriptions of the commercial packages used can be found in their corresponding references. SPM8 [1] is used in MC and AC processing. Comkat [2] and PMOD [3] are used for kinetic modeling. FSL [4] and SPM8 are used for group analysis. Freesurfer [5] is used for regions-of-interest (ROI) definition and smoothing.

*Data preprocessing:* Head-motion is derived from a number of sources: echo-planar MR images, MR-based motion navigators, and directly from the PET data when MR data is unavailable (e.g. during shimming). Subsequently, the ME-MPRAGE is reoriented to the reference position. Cortical and subcortical ROIs are labeled using FreeSurfer; similarly, the MPRAGE is registered to MNI-space for generating subject-specific atlases.

*Image reconstruction:* An OP-OSEM algorithm is used for PET reconstruction [6]. MC [7] and PVC [8] can be performed using the results from data preprocessing. AC can be imported directly from CT, using MR-images [9], or through atlas-based methods.

*Automated Bolus Arrival Time* (*BAT*) *& Image-Derived Input Function:* The singles count rate is recorded during PET acquisition. The BAT is determined by fitting a trilinear piecewise function and used as the reference time. Time-of-Flight MR can then be used to segment the arteries of the head and an image-derived input function can be determined using short frames.

We presented a novel pipeline which interfaces with a number of different commercial software to provide improved PET data quantification.
